# Sensory Reactivity in Children Referred for Autism Evaluation: Associations with Autism Symptoms and Adaptive Skills

**DOI:** 10.3390/brainsci16030310

**Published:** 2026-03-14

**Authors:** Girija Kadlaskar, Stephanie E. King, Jessica R. Stewart

**Affiliations:** Speech Pathology and Audiology, School of Medicine, University of Nevada, Reno, NV 89557, USA; stephanieking@med.unr.edu (S.E.K.); jstewart@med.unr.edu (J.R.S.)

**Keywords:** autism, sensory reactivity, adaptive skills, neurodevelopmental disorders, clinical referrals, sensory profile-2

## Abstract

**Highlights:**

**What are the main findings?**

- Sensory differences are evident across developmental conditions.
- Sensory differences predict adaptive functioning in children with and without autism in clinically referred populations.

**What are the implications of the main findings?**

- Understanding sensory differences across developmental conditions has implications for differential diagnosis.
- Results highlight the importance of examining sensory behaviors in children with diverse clinical profiles.

**Abstract:**

Background: The present study examines sensory differences in children referred for autism evaluations and explores associations between sensory differences, autism symptomatology, and adaptive skills. Using a clinically referred sample, this study captures the heterogeneity of diverse developmental profiles observed in everyday clinical practice and provides a nuanced understanding of sensory differences in an ecologically valid way in the context of autism assessments. Methods: Participants included 238 children (41 females/3–14 years), referred to a university-based autism clinic due to concerns related to autism. Autism diagnoses were confirmed using the Autism Diagnostic Observation Schedule-2, DSM-5 criteria, and expert clinical judgement informed by comprehensive multidisciplinary evaluation. Additional measures were collected to obtain information on sensory processing (Sensory Profile-2/SP-2) and adaptive functioning (Vineland-II/-3). Diagnostic outcomes were classified as autism (*n* = 121) versus non-autism (*n* = 117). Results: Non-autistic children scored higher than autistic children in sensory avoiding and sensitivity, with no group differences in sensory seeking or registration as measured by the SP-2. Correlational analysis showed negative associations between sensory differences and both autism symptomatology and adaptive functioning. Regression analysis further indicated that higher sensory differences predicted lower adaptive functioning, with sensory sensitivity showing the most widespread associations across communication, daily living skills, and socialization. Conclusions: Non-autistic children exhibited greater sensory avoiding and sensitivity than autistic children, which may possibly reflect co-occurring concerns such as anxiety or attentional difficulties (e.g., avoiding noisy environments due to anxiety rather than sensory sensitivity). Across groups, higher sensory differences showed consistent associations with lower adaptive functioning, highlighting the importance of assessing sensory behaviors in children with diverse clinical profiles.

## 1. Introduction

Autism spectrum disorder (autism) is a complex neurodevelopmental condition characterized by differences in social communication and interaction and the presence of restricted and repetitive behaviors [1]. According to the latest edition of the Diagnostic and Statistical Manual of Mental Disorders (DSM-5), differences in sensory processing and reactivity are one of the core diagnostic features of autism, including hyper-reactivity, hypo-reactivity, and sensory seeking in response to surrounding stimuli [1]. These sensory differences have been observed across multiple modalities [2,3,4], and research suggests that the majority of autistic individuals exhibit one or more of these sensory differences [5].

One widely used framework to conceptualize sensory differences is the Dunn’s Model of Sensory Processing that describes sensory behaviors based on the interaction between an individual’s neurological threshold and self-regulation [6]. Neurological thresholds refer to one’s sensitivity for noticing and responding to surrounding sensory inputs. For instance, individuals with a low sensory threshold perceive and respond to sensory stimuli more readily, whereas those with a high sensory threshold may miss sensory cues that others may detect more easily. Self-regulation refers to behavioral strategies that one uses to manage sensory input. For example, an active strategy may involve adjusting one’s behavior to maintain a manageable amount of sensory input, whereas a passive strategy would involve taking minimal actions to manage sensory experiences. Using this model, Dunn [7] delineated sensory processing into four quadrants: seeking (the extent to which one actively engages with sensory input), avoiding (the degree to which one is bothered by sensory input), sensitivity (how easily one detects sensory input), and registration (how often one misses sensory input; [6,7]).

Empirical studies have shown that autistic children and adults show elevated sensory differences across the four sensory quadrants as measured by the child and adult versions of the Sensory Profile questionnaire [8,9,10,11,12,13]. Results indicating greater sensory differences in autism are corroborated by other studies using a variety of sensory questionnaires [14,15], as well as behavioral and electrophysiological paradigms [2,16,17,18]. These differences are clinically meaningful not only because they are a core feature of autism, but also because they impact how individuals engage with their environment, mediating both symptom expression and adaptive skills. For example, several studies have shown that autistic individuals who exhibit higher sensory differences show a greater severity of autism symptoms and lower adaptive skills [19,20].

Notably, differences in sensory processing and/or reactivity are not unique to autism. Children with other developmental conditions—such as Attention-Deficit/Hyperactivity Disorder, Developmental Delay, Language Disorder—may also show elevated sensory differences [21,22,23,24]. Examination of sensory behaviors across these clinical populations is critical for identifying sensory patterns that are unique to autism versus those that are shared across other developmental conditions, thereby facilitating differential diagnosis.

Most studies examining sensory behaviors in neurodevelopmental conditions have largely focused on samples recruited in laboratory settings. While these studies have provided valuable insights, they often include relatively homogenous samples (e.g., younger siblings of children with autism, children with IQ scores above a particular threshold due to the complexity of tasks being completed etc.) [4,15,16]. As a result, findings may not fully capture the heterogeneity of sensory behaviors often encountered in everyday clinical practice. The present study aims to address this gap in the literature by examining sensory behaviors in a sample of children specifically referred for autism evaluations. Such an approach offers several advantages. First, it provides ecologically valid insights into how sensory differences manifest in clinical settings among children with diverse developmental and family backgrounds, where multiple neurodevelopmental and/or behavioral concerns may be evident. Second, this approach enables the examination of how sensory differences may relate to autism symptomatology and adaptive skills in a clinically diverse sample, highlighting patterns that may be relevant to assessment and intervention in everyday clinical practice.

This study had 3 aims: (1) to examine group differences in caregiver-reported sensory reactivity, as measured by Dunn’s four sensory quadrants, in a sample of school-aged children referred for autism evaluations at a university-based clinic; (2) to examine associations between sensory reactivity, autism symptomatology, and adaptive skills; and (3) to assess whether sensory behaviors predict autism symptomatology and adaptive skills in all children. We hypothesized that autistic children would show elevated sensory differences across the four quadrants compared to the non-autistic group and that the sensory behaviors would be associated with and account for variance in autism symptomatology and adaptive skills in all children.

## 2. Methods

### 2.1. Participants

A total of 394 children and adolescents (2–18 years) were evaluated at a University of Nevada, Reno-based autism clinic (University Center for Autism and Neurodevelopment; UCAN) between 2014 and 2024 due to concerns related to autism. The present study conducted a retrospective analysis of archival medical records from these evaluations. The study protocol was reviewed and approved by the University of Nevada, Reno Institutional Review Board (IRB #2282771), which granted a waiver of informed consent for this study due to the retrospective nature of the archival data analysis and the impracticability of obtaining consent. All procedures were conducted in accordance with the ethical standards of the Belmont Report and the regulations of the 2018 revised Common Rule.

### 2.2. Procedure at the Time of Autism Evaluation

Typical procedures of care at the university-based clinic consisted of being referred for an autism evaluation. Referral sources originated from pediatricians, community providers, educational staff, or self-referrals. As part of the intake process, families were contacted to complete a parent/caregiver interview that included questions regarding the child’s development and behavior. Additionally, families were requested to submit any medical and educational records for the child. If the child was found to be an appropriate candidate to receive an autism evaluation based on referral information, the family was then scheduled for a series of assessments and interviews that included autism-specific structured interviews (ADI-R; [25]), adaptive behavior assessments (Vineland-II; Vineland-3; [26,27]), parent/caregiver-reported sensory questionnaires (Sensory Profile-2; [28]), behavioral observations, and language and cognitive assessments (Clinical Evaluation of Language Fundamentals, Test of Problem Solving, Peabody Picture Vocabulary Test, Expressive Vocabulary Test, Mullen Scales of Early Learning, Wechsler Intelligence Scale for Children; [29,30,31,32,33,34]). Additionally, when applicable, questionnaires were sent to the child’s teachers to gain a comprehensive overview of the child across settings. Note that not all children completed every assessment; the specific measures administered were determined based on both the prior availability of testing and the individual needs of the child. Data related to the family’s income, parental education, and racial and ethnic background were not collected as part of the evaluations.

After completing caregiver questionnaires, interviews, behavioral observations, and cognitive and language assessments, the families returned for a second clinic visit to complete the ADOS-2 [35]. The ADOS-2 was administered by a trained clinician while a multidisciplinary team—consisting of a pediatrician, child and adolescent psychiatrist, psychologist, speech-language pathologist, occupational therapist, marriage and family counselor, and a developmental specialist—observed the session live via a monitor in an adjacent room. After the completion of the ADOS-2, the administrator joined the UCAN team in the adjacent room for a consensus coding session, during which each team member independently scored the ADOS-2 and shared their item-level ratings. In cases of disagreements, the team discussed the rationale behind the scores in question and reached a consensus based on majority agreement. In addition to the ADOS-2 scores, the multidisciplinary team reviewed previous medical and academic records, current assessment data, and reviewed the diagnostic criteria for autism spectrum disorder based on the DSM-5 [1]. Considering all of the testing data, family and teacher reports, and the DSM-5, an informed decision was made as to whether the child met or did not meet the diagnostic criteria for a diagnosis of autism spectrum disorder. All families received a detailed clinical report outlining the diagnostic outcomes for their child. If concerns were raised about the possibility of a diagnosis in addition to, or instead of, autism, recommendations were made for further assessments.

### 2.3. Procedure for Archival Data Analysis

Given the scope of the present study, inclusion in the archival data analysis required (1) availability of the Sensory Profile-2 data, (2) a documented diagnostic outcome based on the final clinical report provided by the UCAN team, and (3) availability of at least one of the two standardized diagnostic measures: ADOS-2 [35] and Vineland Adaptive Behavior Scales-II/3 (Vineland-II for children assessed before 2016 and Vineland-3 for children assessed from 2016 onward; [26,27]). This resulted in a final sample of 238 children, with only 1 child missing scores for the Vineland-3. Based on the information provided in clinical reports, diagnostic outcomes for the 238 children were classified as autism (*n* = 121) versus non-autism (*n* = 117). Out of 238, 142 children (47 non-autistic) had other diagnoses such as ADHD, language disorders, anxiety, intellectual disability, unspecified neurodevelopmental disorder, obsessive compulsive disorder (OCD), etc., based on prior testing conducted elsewhere (Tables S1 and S2 in the Supplementary Materials).

Sensory Profile-2 quadrant scores and ADOS-2 comparison scores were extracted from scanned copies of the scored protocols stored in the clinic database. Standard scores for the Vineland-II and Vineland-3 were obtained from the clinical reports provided to families following their evaluations and securely stored in the clinic database. See Table 1 for the participant characteristics.

### 2.4. Measures

#### 2.4.1. Sensory Profile-2 (SP-2)

The Sensory Profile 2 is a parent/caregiver questionnaire that assesses everyday sensory reactivity in 3-to-14-year-old children [28]. The SP-2 provides scores across six sensory domains (auditory, visual, tactile, movement, body position, and oral), three behavioral domains (conduct, social emotional, and attentional), and four sensory quadrants (seeking, avoiding, sensitivity, and registration). The four sensory quadrant scores were used as a measure of sensory reactivity, with high scores indicating that the behavior in question is observed more frequently compared to children in the same age range.

#### 2.4.2. Autism Diagnostic Observation Schedule-2 (ADOS-2)

The Autism Diagnostic Observation Schedule, 2nd Edition (ADOS-2) is a standardized, semi-structured assessment of communication, social interaction, play, and restricted and repetitive behaviors [35]. Children were administered Modules 1–4 based on the module-specific language-level requirements as per the ADOS-2 manual. ADOS-2 comparison scores were used as a measure of the severity of autism characteristics, with high scores indicating greater autism symptomatology.

#### 2.4.3. Vineland-II/Vineland-3

The Vineland Adaptive Behavior Scales-2nd ed/The Vineland-3 is a caregiver questionnaire/interview designed to examine adaptive functioning in four domains: communication, daily living skills, socialization, and motor skills [26,27]. All participants received the Vineland Comprehensive interview form. The Vineland-II was administered to parents/caregivers of children evaluated before 2016, and Vineland-3 was used from 2016 onward. Standard scores for the communication, daily living skills, and socialization domains were used in the present analysis, with high scores indicating greater adaptive functioning.

### 2.5. Statistical Approach

Independent-samples *t* tests were conducted to examine group differences in SP-2 quadrant scores between autistic (*n* = 121) and non-autistic (*n* = 117) children. Correlations were conducted to examine associations between SP-2 quadrant scores and autism symptomatology and adaptive skills as measured by the ADOS-2 and the Vineland-II and -3, respectively. Given that this dataset was collected over multiple years as part of clinical assessments, some children (*n* = 81) were assessed using the Vineland-II, while others (*n* = 156) were assessed using the Vineland-3, with one child missing the Vineland data. Because Vineland-3 includes revised items, updated scoring, and changes to domain structure, scores from Vineland-II and -3 were not combined or directly compared [36]. Therefore, analyses were conducted separately for each version of the Vineland. Some variables (ADOS-2 comparison scores, Vineland-3 domain scores) were non-normally distributed, and data transformations did not normalize the distributions. Thus, Spearman rank correlations were used for non-normally distributed variables, while Pearson correlations were used for normally distributed variables (Vineland II domain scores). Finally, multiple regression analysis was conducted to examine whether sensory quadrant scores predicted autism symptomatology as well as adaptive skills. Age and diagnostic status were included as covariates in the regression models based on previous research showing age- and diagnosis-related differences in sensory processing in autistic individuals [37]. Finally, given the large number of associations across measures in the regression analysis, we restricted the alpha level to *p* < 0.01 to reduce the likelihood of Type I error. We elected to report both correlations and regression in the present study as the two analyses address related but distinct questions and provide a more comprehensive understanding of the data; correlations show patterns of associations between SP-2 quadrant scores and outcomes, while regression analysis extends these results by assessing the unique contributions of sensory behaviors on autism symptomatology and adaptive skills after accounting for potential confounding variables. All analyses were conducted in SPSS version 31.0.

## 3. Results

### 3.1. Group Differences in Sensory Profiles

**Figure 1:**
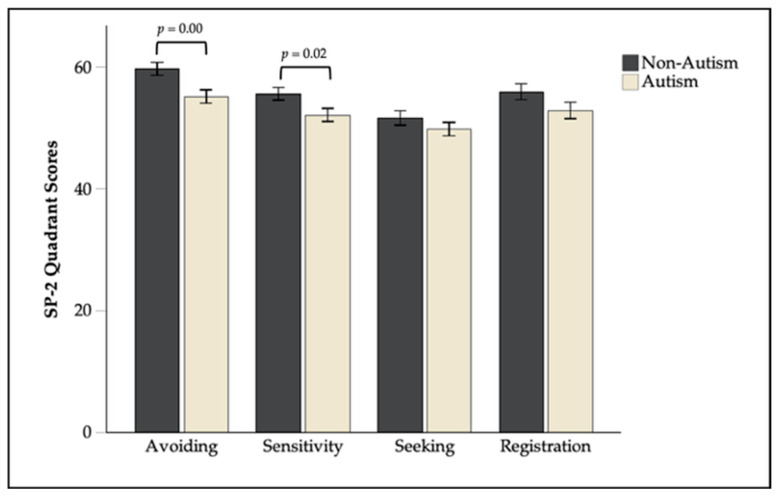
Mean Sensory Profile-2 (SP-2) quadrant scores by diagnostic group. Error bars represent ± 1 standard error.

T-tests showed that non-autistic children scored higher than autistic children on avoiding (non-autistic: *M* = 59.69, *SD* = 11.60; autistic: *M* = 55.15, *SD* = 12.16), *t*(235.96) = 2.95, *p* = 0.00, *d* = 0.38, 95% CI [0.13, 0.64] and sensitivity (non-autistic: *M* = 55.62, *SD* = 11.35; autistic: *M* = 52.14, *SD* = 11.62), *t*(235.98) = 2.34, *p* = 0.02, *d* = 0.30, 95% CI [0.05, 0.56]. No significant group differences were observed for seeking (non-autistic: *M* = 51.66, *SD* = 12.78; autistic: *M* = 49.80, *SD* = 12.27), *t*(234.69) = 1.14, *p* = 0.25, *d* = 0.15, 95% CI [−0.11, 0.40] or registration (non-autistic: *M* = 55.93, *SD* = 14.11; autistic: *M* = 52.85, *SD* = 14.39), *t*(235.96) = 1.67, *p* = 0.10, *d* = 0.22, 95% CI [−0.04, 0.47], Figure 1; Table 2).

### 3.2. Correlations

For all children, avoiding was negatively correlated with ADOS-2 comparison scores (r_s_ (219) = −0.18, *p* = 0.01), indicating that higher sensory avoiding was associated with lower autism symptom severity. In terms of adaptive functioning, registration was negatively associated with daily living skills on the Vineland-II (r (81) = −0.25, *p* = 0.03). For Vineland-3, higher SP-2 quadrant scores were generally associated with lower adaptive functioning. Specifically, seeking was negatively correlated with daily living skills (r_s_ (155) = −0.18, *p* = 0.02) and socialization (r_s_ (156) = −0.23, *p* = 0.00). Avoiding was negatively correlated with socialization (r_s_ (156) = −0.32, *p* < 0.001). Sensitivity and registration were negatively correlated with communication, daily living skills, and socialization (Table 3). These results suggest that sensory differences are linked to functional outcomes across various adaptive domains, highlighting their relevance for understanding everyday communication, daily living skills, and socialization in children referred for autism evaluations.

### 3.3. Regression

The overall regression model predicting the ADOS-2 comparison score was significant (F (6, 212) = 80.59, *p* < 0.001). However, an examination of the individual predictors revealed that diagnosis was a significant predictor (*p* < 0.001), with none of the SP-2 quadrant scores or age predicting the ADOS-2 comparison score, suggesting that sensory behaviors did not account for additional variance in autism symptomatology beyond diagnostic status.

For Vineland-II, the overall regression model predicting daily living skills was significant (F (6, 74) = 3.17, *p* = 0.00), with registration being negatively associated with daily living skills (B = −0.51, *p* = 0.00). These results indicate that children who were more likely to miss surrounding sensory input showed lower daily living skills as measured by the Vineland-II. The SP-2 quadrant scores did not predict Vineland-II communication and socialization skills (all *ps* > 0.06).

Next, the SP-2 quadrant scores predicted all three domains of the Vineland-3: communication, daily living skills, and socialization. Examination of individual predictors for each model showed that sensitivity (B = −0.61, *p* = 0.00) and diagnostic status (B = −9.25, *p* = 0.00) significantly predicted communication scores. Seeking (B = 0.31, *p* = 0.00), sensitivity (B = −0.65, *p* < 0.001), age (B = 0.87, *p* = 0.00), and diagnostic status (B = −7.21, *p* < 0.001) significantly predicted daily living skills, while sensitivity (B = −0.40, *p* = 0.00) and diagnostic status (B = −14.62, *p* < 0.001) significantly predicted socialization (Table 4). Overall, these results suggest that higher caregiver-reported sensitivity to surrounding sensory stimuli was consistently associated with lower adaptive functioning in all children across communication, daily living skills, and socialization as measured by the Vineland-3. In contrast, higher seeking behaviors predicted higher daily living skills, indicating that children who actively seek sensory input may show higher everyday functioning, at least in some instances.

## 4. Discussion

This study aimed to examine caregiver-reported sensory behaviors in a sample of school-aged children referred for an autism evaluation at a university-based autism clinic. Furthermore, the study also assessed associations between sensory behaviors, autism symptomatology, and adaptive skills. Contrary to our expectations, non-autistic children exhibited greater sensory avoiding and sensitivity than autistic children as per caregiver report, indicating that sensory differences are not unique to autism and are evident across clinically referred populations. The two groups did not differ in terms of sensory seeking and registration. Our results are consistent with prior research highlighting sensory differences in other developmental conditions [38,39,40], but they contrast with studies reporting higher sensory differences in autistic individuals compared to those with other developmental conditions [41,42].

Importantly, our findings should be interpreted within the context of a clinically referred sample, as all children included in the study were evaluated for developmental and/or behavioral concerns as reported by caregivers, pediatricians, teachers, and/or other professionals. As such, many children in the non-autistic group presented with one or more behavioral concerns or diagnoses, including language disorder, ADHD, intellectual disability, social communication difficulties, anxiety, developmental delay, or, in some cases, a deferred diagnosis. While sensory differences are observed in many of these conditions, such as ADHD, developmental delay etc. [21,41,43], within this clinically referred sample, higher avoiding and sensitivity among the non-autistic group may also reflect behavioral or regulatory difficulties rather than sensory-specific differences that are characteristic of autism. For instance, several items in the SP-2 that contribute to the avoiding and sensitivity quadrants (e.g., showing predictable fears, having temper tantrums, becoming easily frustrated, being distracted by background noise, distress during grooming activities, gets lost easily etc.) may be influenced by broader concerns such as anxiety, attentional difficulties, or emotion regulation challenges that were evident in many children in the non-autistic group. This referral bias may have contributed to our findings, potentially leading to higher scores on at least some of the SP-2 items. These results emphasize the importance of considering clinical context, referral bias, and co-existing conditions while assessing sensory behaviors in clinical settings.

Another possible explanation for these results could be attributed to different coping mechanisms that children may use to manage overwhelming sensory input. Engaging in avoidance behaviors may serve as a strategy to cope with stress and anxiety in sensory overwhelming situations, which in turn can support better focus on tasks at hand. A recent study by Shulman and colleagues [44] showed that autistic children with greater cognitive functioning exhibited more sensory avoidance compared to autistic children with lower cognitive abilities, highlighting strategies that some children may use to manage intense sensory experiences. It is possible that the non-autistic group in the present study may have similarly employed sensory avoidance as a strategy to support functioning across other areas of daily life. Future research should examine whether observed patterns reflect true sensory processing differences or represent overlapping behavioral challenges or strategies that mimic sensory features in heterogeneous clinical populations. Additionally, clinicians and researchers should explore the utility of additional sensory questionnaires, such as the Sensory Experiences Questionnaire [14], alongside the SP-2 in clinical settings, to better understand sensory differences and to examine their potential relevance to autism diagnostic outcomes in children referred for evaluation.

Next, sensory avoiding was negatively associated with ADOS-2 comparison score, suggesting that higher levels of avoiding were linked to lower autism symptom severity. This may indicate the same phenomenon discussed above for group differences. Sensory avoidance behaviors may reflect co-occurring traits such as anxiety or self-regulation differences, rather than greater severity of autism symptoms, particularly in this clinically referred sample, where such concerns were commonly reported. Past research has shown links between sensory differences, anxiety, and self-regulation in autistic and clinically referred children [38,45,46]. While examining the mechanisms underlying the association between higher sensory avoiding and lower autism symptoms was beyond the scope of this study, these findings provide a rationale for future research examining how sensory behaviors may interact with co-existing conditions often observed in clinical populations.

As expected, our results showed that sensory behaviors were correlated with adaptive functioning, particularly as measured by the Vineland-3. In line with prior research, children showing higher sensory seeking, avoiding, sensitivity, or registration exhibited lower scores across communication, daily living skills, and socialization [20,47]. Notably, these associations were more consistently observed in the Vineland-3 compared to the Vineland-II. Several factors may have contributed to this difference. The Vineland-3 includes several updated items and revised domain structures that may more comprehensively capture one’s adaptive skills. Moreover, recent research has shown that individuals with intellectual and developmental disabilities generally score lower on the Vineland-3 compared to Vineland-II, and these differences are larger for individuals with lower levels of ability [36]. This suggests that in the present sample, the Vineland-3 may have been more sensitive to detect associations between sensory behaviors and adaptive functioning than the Vineland-II.

Our regression analysis showed that while sensory differences did not predict autism symptomatology, they did predict adaptive skills, controlling for age and diagnostic status. Specifically, higher sensory registration predicted lower daily living skills for the Vineland-II. Extending these findings, sensory differences predicted all three domains of the Vineland-3: communication, daily living skills, and socialization. Aligning with past research, higher sensitivity predicted lower communication, daily living skills, and socialization [20,48,49]. As expected, the autistic group was more likely to show lower communication, daily living, and socialization skills, and older children were more likely to show higher daily living skills, reflecting developmental gains in adaptive functioning.

Sensory seeking, however, exhibited a more complex association with adaptive skills. While higher seeking behavior was correlated with lower daily living skills and socialization, our regression analysis, which controlled for co-occurring sensory traits along with age and diagnostic status, showed that higher seeking behaviors predicted higher daily living skills. These results could be attributed to the structure of the SP-2. For instance, in our sample, the four SP-2 quadrants were positively correlated, and when controlling for the shared variance across quadrants, some aspects of sensory seeking may support daily living skills. This aligns with previous research suggesting that sensory seeking may play a facilitating role in early social development [50]. Specifically, sensory seeking behaviors may lead to more object exploration, thereby creating opportunities for practicing daily living skills. While we acknowledge that sensory seeking may not *always* be beneficial [24,51], these results suggest that seeking behaviors may have the potential to support daily living skills at least in some cases. However, more research is warranted to examine this hypothesis in further detail.

Our study is not without limitations. First, because the data were collected as part of an autism clinic at the University of Nevada, Reno, key demographic information such as racial and ethnic background, parental education, and family income was not collected at the time of evaluation. This limited our ability to explore sensory differences based on various demographic factors, and as a result, may impact the generalizability of our findings. Second, sensory behaviors were measured via caregiver report. While caregiver report provides valuable information about children’s sensory behaviors across everyday contexts, these reports may be subjective and influenced by caregiver perceptions or expectations. Third, given that clinic evaluations included both the Vineland-II and Vineland-3, adaptive behavior scores could not be pooled across all children, requiring separate analysis. This may have impacted the statistical power of our results. Finally, the non-autistic group included children who did not meet the criteria for autism, but many did exhibit a range of one or more behavioral and/or developmental conditions as per prior clinical reports. Given the heterogeneity of diagnoses in the non-autistic group (e.g., ADHD, language disorder, anxiety, intellectual disability, speech difficulties, OCD, deferred diagnosis etc.), analyses within these individual conditions could not be conducted due to limited power.

## 5. Conclusions

In sum, non-autistic children exhibited greater sensory avoiding and sensitivity than autistic children, highlighting that sensory behaviors are not unique to autism. Within clinically referred populations, such patterns may reflect other developmental or behavioral concerns, like anxiety or attentional difficulties (e.g., avoiding noisy settings due to anxiety rather than sensory sensitivity), or they may also represent a coping mechanism to manage overwhelming sensory input. Future research should examine whether observed patterns reflect true sensory processing differences or represent overlapping behavioral challenges that mimic sensory features in this population. Finally, higher sensory differences were associated with lower adaptive functioning, with sensory sensitivity showing the most widespread associations across communication, daily living skills, and socialization. These results emphasize the clinical relevance of assessing sensory behaviors in diverse developmental profiles.

## Figures and Tables

**Table 1:** Participant Characteristics.

	Autism (*n* = 121)	Non-Autism (*n* = 117)	Statistic	*p*-Value
Age, mean (SD)	7.01 (2.92)	8.56 (3.06)	*t*(236) = 3.98	<0.001 **
Gender, *n* (%)			X^2^(1) = 1.74	0.18
Female	17 (14%)	24 (21%)		
Male	104 (86%)	93 (79%)		
ADOS-2 Modules ^a^			X^2^(3) = 50.28	<0.001 **
Module 1	37 (32.2%)	2 (1.9%)		
Module 2	32 (27.8%)	17 (16.3%)		
Module 3	46 (40%)	79 (76.0%)		
Module 4	-	6 (5.8%)		
ADOS-2, mean (SD) ^a^				
Comparison Score	7.88 (1.62)	2.41 (2.04)	*t*(217) = −22.02	<0.001 **
Vineland-II mean (SD)				
*n* = 81	30	51		
Communication	68.50 (16.76)	77.06 (11.27)	*t*(79) = 2.74	0.01 *
Daily Living Skills	76.17 (13.51)	81.47 (13.54)	*t*(79) = 1.70	0.09
Socialization	69.07 (10.92)	71.16 (11.42)	*t*(79) = 0.80	0.42
Vineland-3 mean (SD) ^b^				
*n* = 156	90	66		
Communication	63.74 (20.90)	73.36 (15.16)	*t*(154) = 3.17	0.00 **
Daily Living Skills	74.08 (13.29)	81.29 (12.00)	*t*(153) = 3.47	<0.001 **
Socialization	61.18 (15.96)	73.27 (14.32)	*t*(154) = 4.88	<0.001 **

Note. ADOS-2, Autism Diagnostic Observation Schedule-2. ^a^ ADOS-2 scores were missing for 6 children in the autism group and 13 children in the non-autism group. ^b^ Vineland-3 scores were missing for one child in the autism group. * *p* < 0.05; ** *p* < 0.01.

**Table 2:** Group differences in Sensory Profile-2 quadrant scores.

	Autism		Non-Autism		*t*	*p*	Cohen’ d
	*M*	*SD*	*M*	*SD*			
Avoiding	55.15	12.16	59.69	11.60	2.95	0.00 **	0.38
Sensitivity	52.14	11.62	55.62	11.35	2.34	0.02 *	0.30
Seeking	49.80	12.27	51.66	12.78	1.14	0.25	0.15
Registration	52.85	14.39	55.93	14.11	1.67	0.10	0.22

* *p* < 0.05; ** *p* < 0.01.

**Table 3:** Correlations between Sensory Profile-2 sensory quadrants, autism symptomatology, and adaptive skills.

	Seeking	Avoiding	Sensitivity	Registration
ADOS-2 comparison score ^#^ (*n* = 219)	−0.04	−0.18 **	−0.12	−0.10
Vineland-II ^ (*n* = 81)				
Communication	0.01	0.17	0.11	0.00
Daily Living Skills	−0.15	0.10	−0.03	−0.25 *
Socialization	−0.05	0.05	−0.07	−0.17
Vineland-3 ^#^ (*n* = 156)				
Communication	−0.06	−0.06	−0.21 **	−0.18 *
Daily Living Skills	−0.18 *	−0.14	−0.34 **	−0.029 **
Socialization	−0.23 **	−0.32 **	−0.36 **	−0.34 **

Note. ^#^ Spearman’s rho correlations; ^ Pearson correlations. ADOS-2, Autism Diagnostic Observation Schedule-2. * *p* < 0.05; ** *p* < 0.01.

**Table 4:** Regression analysis of SP-2 quadrant scores, autism symptomatology, and adaptive skills.

	Predictor	B	SE B	β	*t*	*p*
ADOS-2 comparison score (*n* = 219) ^a^	Seeking	0.01	0.02	0.03	0.48	0.63
	Avoiding	−0.02	0.02	−0.08	−1.26	0.21
	Sensitivity	0.01	0.02	0.02	0.29	0.77
	Registration	0.00	0.01	0.01	0.30	0.82
	Age	0.06	0.05	0.06	1.37	0.17
	Dx status	5.51	0.26	0.89	21.23	<0.001 **
Vineland-II (*n* = 81)						
Communication ^b^	Seeking	−0.21	0.18	−0.18	−1.17	0.25
	Avoiding	0.36	0.22	0.28	1.61	0.11
	Sensitivity	0.12	0.24	0.09	0.49	0.63
	Registration	−0.16	0.17	−0.14	−0.94	0.35
	Age	−0.91	0.61	−0.19	−0.19	0.14
	Dx status	−9.43	3.41	−0.33	−2.77	0.00 **
Daily Living Skills ^c^	Seeking	−0.18	0.17	−0.16	−1.05	0.30
	Avoiding	0.40	0.21	0.32	1.93	0.06
	Sensitivity	0.14	0.22	0.10	0.61	0.55
	Registration	−0.51	0.16	−0.46	−3.15	0.00 **
	Age	0.19	0.58	0.04	0.32	0.75
	Dx status	−5.12	3.20	−0.18	−1.60	0.11
Socialization ^d^	Seeking	−0.02	0.15	−0.03	−0.16	0.88
	Avoiding	0.30	0.18	0.30	1.66	0.10
	Sensitivity	−0.11	0.20	−0.11	−0.58	0.57
	Registration	−0.24	0.14	−0.27	−1.72	0.09
	Age	−0.28	0.51	−0.08	−0.56	0.58
	Dx status	−2.72	2.81	−0.12	−0.97	0.34
Vineland-3 (*n* = 156)						
Communication ^e^	Seeking	0.34	0.16	0.23	2.09	0.04
	Avoiding	0.25	0.18	0.16	1.44	0.15
	Sensitivity	−0.61	0.20	−0.38	−3.02	0.00 **
	Registration	−0.24	0.16	−0.19	−1.53	0.13
	Age	0.76	0.51	0.12	1.50	0.14
	Dx status	−9.25	2.99	−0.24	−3.09	0.00 **
Daily Living Skills ^f^	Seeking	0.31	0.10	0.30	3.06	0.00 **
	Avoiding	0.16	0.11	0.14	1.42	0.16
	Sensitivity	−0.66	0.13	−0.59	−5.20	<0.001 **
	Registration	−0.15	0.10	−0.17	−1.57	0.12
	Age	0.88	0.32	0.21	2.78	0.00 **
	Dx status	−7.21	1.89	−0.27	−3.82	<0.001 **
Socialization ^g^	Seeking	0.16	0.12	0.13	1.34	0.18
	Avoiding	−0.23	0.13	−0.18	−1.78	0.08
	Sensitivity	−0.40	0.15	−0.30	−2.69	0.00 **
	Registration	−0.14	0.12	−0.13	−1.19	0.24
	Age	−0.49	0.37	−0.09	−1.32	0.19
	Dx status	−14.62	2.22	−0.44	−6.59	<0.001 **

Note. B = unstandardized coefficient; SE B = standard error; β = standardized coefficient; *t* = *t*-value; *p* = significance. ADOS-2, Autism Diagnostic Observation Schedule-2; Dx status, diagnostic status. ^a^ R^2^ = 0.70; ^b^ R^2^ = 0.15; ^c^ R^2^ = 21; ^d^ R^2^ = 0.08; ^e^ R^2^ = 0.17; ^f^ R^2^ = 0.32; ^g^ R^2^ = 0.32. ** *p* < 0.01.

## Data Availability

The data presented in this study are available on request from the corresponding author due to privacy and ethical reasons, and require approval from the institution and the ethics committee.

## Supplementary Materials

The following supporting information can be downloaded at: https://www.mdpi.com/article/10.3390/brainsci16030310/s1, Table S1. Diagnostic classification of the sample (N = 238). Table S2. Frequency of additional diagnosis/conditions by diagnostic status.
