# Supplementary material for: Sensory Reactivity in Children Referred for Autism Evaluation: Associations with Autism Symptoms and Adaptive Skills
Source: Brain Sci. 2026 Mar 14;16(3):310. doi: 10.3390/brainsci16030310 (PMC13025295; doi:10.3390/brainsci16030310)
Supplement: Supplementary file 1 [file brainsci-16-00310-s001.zip › brainsci-4161395-supplementary.pdf]

Table S1. Diagnostic classification of the sample (N = 238)

Diagnostic group	*n*	Percentage of sample
Autism only	49	40%
Autism with one or more diagnosis	72	60%
Non-Autism with deferred diagnosis	47	40%
Non-Autism with one or more diagnosis	70	60%

Table S2. Frequency of additional diagnosis/conditions by diagnostic status.

Additional diagnoses/conditions	Autism with one or more additional diagnosis (n = 72)	Non-Autism with one or more additional diagnosis (n = 70)
ADHD	7	19
Anxiety	2	17
Language Disorder	62	34
Developmental Coordination Disorder	1	5
Intellectual disability	4	5
Oppositional Defiant Disorder	-	1
Unspecified Neurodevelopmental Disorder	-	7
Neglect and Trauma	2	1
Chromosomal abnormality	1	1
Social Pragmatic Disorder	-	3
Fetal Alcohol Syndrome	-	1
Hearing loss	-	1
Articulation Disorder	-	2
Bipolar Disorder	-	1
Speech Sound Disorder	3	1
Mood Disorder	1	1
Obsessive Compulsive Disorder	-	1
Reactive Attachment Disorder	-	2
Unspecified Neurological Disorder	-	1
Depression	1	-
Seizure	1	-
Tic Disorder	1	-
Preterm Birth	1	-

Note. Inclusion of additional diagnostic information was based on parent/caregiver report and/or the availability of prior clinical or educational testing conducted outside of the University autism clinic. Many children were identified as having more than one additional diagnosis; therefore, the sum of diagnoses exceeds the number of children within each group.
